# Complete Genome Sequences of *Peach Latent Mosaic Viroid* from a Single Peach Cultivar

**DOI:** 10.1128/genomeA.01098-15

**Published:** 2015-09-24

**Authors:** Yeonhwa Jo, Su-Hyun Yoo, Hyosub Chu, Jin Kyong Cho, Hoseong Choi, Ju-Yeon Yoon, Seung-Kook Choi, Won Kyong Cho

**Affiliations:** a Department of Agricultural Biotechnology, College of Agriculture and Life Sciences, Seoul National University, Seoul, Republic of Korea; b Department of Fruit Tree, Korea National College of Agriculture and Fisheries, Jeonju, Republic of Korea; c Department of Horticultural Environment, Virology Unit, National Institute of Horticultural and Herbal Science, RDA, Wan-Ju, Republic of Korea

## Abstract

*Peach latent mosaic viroid* (PLMVd) is a member of the genus *Pelamoviroid* in the family *Avsunviroidae* and infects peach trees. We *de novo* assembled a PLMVd genome from a peach transcriptome and identified 18 variants in a single peach cultivar, after sequencing 20 PLMVd genomes by Sanger sequencing.

## GENOME ANNOUNCEMENT

Viroids are the smallest pathogen composed of a circular single-stranded RNA, which does not encode any protein (1, 2). The known hosts for viroids are limited to plants. Of known viroids, *Peach latent mosaic viroid* (PLMVd) is a member of the genus *Pelamoviroid* in the family *Avsunviroidae* (3, 4). So far, it is known that PLMVd infects *Prunus* species, including apricot, cherry, pear, nectarine, plum, peach, and peach hybrids (5). In general, infection of PLMVd does not lead to disease symptoms; however, some pathogenic PLMVds cause mosaic and white patterns (peach calico), chlorotic mosaic, and peach foliage in the infected leaves (6). PLMVds have been identified in many peach-growing countries and are usually transmitted by grafting and aphids (*Myzus persicae*); however, no seed transmission has yet been reported (3).

To identify viruses and viroids infecting peach trees, we sampled leaves of many different peach cultivars from diverse peach orchards in May 2014, in the Republic of Korea. We extracted total RNA from each peach cultivar and generated libraries for RNA-Seq using the TruSeq RNA Library prep kit version 2 (Illumina, USA) according to the manufacturer’s instructions. Libraries were paired-end sequenced using Illumina’s HiSeq 2000 platform in Macrogen, Republic of Korea. We *de novo* assembled each peach transcriptome using the Trinity program (7). The obtained individual transcriptome was blasted against viral reference genomes to identify viruses and viroids infecting peach. All examined peach transcriptomes include sequences of PLMVd. Among them, a transcriptome derived from the cultivar “Cheonjungdo” contains a complete PLMVd genome sequence, which was *de novo* assembled. We named the newly identified isolate as PLMVd isolate “Cheonjungdo” based on the identified peach cultivar. To confirm the assembled PLMVd genome sequence by Sanger sequencing, we conducted RT-PCR using PLMVd-specific primers (5′ AACTGCAGTGCTCCGAATAGGGCAC 3′/5′ CCCGATAGAAAGGCTAAGCACCTCG 3′), which amplify the complete genome of PLMVd. The PCR products were cloned, and a total of 20 clones were sequenced by Sanger sequencing. Of the 20 newly sequenced PLMVd genomes, we identified 18 variants. The genome sizes of 20 PLMVds ranged from 337 to 339 nucleotides. A BLAST search found that the PLMVd isolate “Cheonjungdo” was closely related to the known PLMVd isolate ZZ30 from China (NCBI accession number JF898816). The obtained PLMVd sequences were combined to generate a consensus PLMVd genome sequence in cultivar “Cheongjungdo.” To reveal single nucleotide variations (SNVs) in a single peach tree, we aligned all variant sequences to the consensus PLMVd sequence and identified 77 SNVs.

Taken together, our study successfully applied peach transcriptome data to obtain the PLMVd genome, which was *de novo* assembled. Moreover, we sequenced 20 additional PLMVd genomes in the same cultivar, revealing a quasi-species of PLMVd in a single peach tree.

### Nucleotide sequence accession numbers.

The genome sequences of *Peach latent mosaic viroid* have been submitted to GenBank under the accession numbers KT033033 to KT033052.
